# Multifunctional PLA Blends Containing Chitosan Mediated Silver Nanoparticles: Thermal, Mechanical, Antibacterial, and Degradation Properties

**DOI:** 10.3390/nano10010022

**Published:** 2019-12-20

**Authors:** Agueda Sonseca, Salim Madani, Gema Rodríguez, Víctor Hevilla, Coro Echeverría, Marta Fernández-García, Alexandra Muñoz-Bonilla, Noureddine Charef, Daniel López

**Affiliations:** 1 MacroEng Group, Instituto de Ciencia y Tecnología de Polímeros, ICTP-CSIC, C/ Juan de la Cierva 3, 28006 Madrid, Spain; gema@ictp.csic.es (G.R.); v.hevilla@ictp.csic.es (V.H.); cecheverria@ictp.csic.es (C.E.); martafg@ictp.csic.es (M.F.-G.); sbonilla@ictp.csic.es (A.M.-B.); 2 Interdisciplinary Plataform for “Sustainable Plastics towards a Circular Economy” (SUSPLAST-CSIC), 28006 Madrid, Spain; 3 Instituto de Tecnología de Materiales, Universitat Politècnica de València (UPV), Camino de Vera s/n, 46022 Valencia, Spain; 4 Laboratory of Applied Biochemistry, University Ferhat Abbas, Setif 19000, Algeria; madanisalim79@gmail.com (S.M.); charefnr@hotmail.com (N.C.)

**Keywords:** poly(lactic acid), oligomeric lactic acid, eco-friendly silver nanoparticles, biopolymer properties, antimicrobial activity, packaging

## Abstract

Poly(lactic acid) (PLA) is one of the most commonly employed synthetic biopolymers for facing plastic waste problems. Despite its numerous strengths, its inherent brittleness, low toughness, and thermal stability, as well as a relatively slow crystallization rate represent some limiting properties when packaging is its final intended application. In the present work, silver nanoparticles obtained from a facile and green synthesis method, mediated with chitosan as a reducing and stabilizing agent, have been introduced in the oligomeric lactic acid (OLA) plasticized PLA in order to obtain nanocomposites with enhanced properties to find potential application as antibacterial food packaging materials. In this way, the green character of the matrix and plasticizer was preserved by using an eco-friendly synthesis protocol of the nanofiller. The X-ray diffraction (XRD) and differential scanning calorimetry (DSC) results proved the modification of the crystalline structure as well as the crystallinity of the pristine matrix when chitosan mediated silver nanoparticles (AgCH-NPs) were present. The final effect over the thermal stability, mechanical properties, degradation under composting conditions, and antimicrobial behavior when AgCH-NPs were added to the neat plasticized PLA matrix was also investigated. The obtained results revealed interesting properties of the final nanocomposites to be applied as materials for the targeted application.

## 1. Introduction

Although biodegradable polymers are not meant to fully substitute the non-biodegradable synthetic polymers, when applying them in packaging and medical industries it is still one of the most hopeful approaches to face polymer waste’s environmentally harmful problems [1]. Poly(lactic acid) (PLA) is a well-known biodegradable synthetic polymer that is considered one of the most attractive materials for packaging applications [2] thanks to its biocompatibility, biodegradability, renewable character (can be obtained from the fermentation of 100% renewable and biodegradable plant sources such as corn, rice, and sugar feedstocks), as well as commercial availability [3,4]. PLA has also been approved by the American Food and Drug Administration (FDA) for direct contact with biological fluids, and it is also highly transparent and possesses good water vapor barrier properties [5,6]. In general, PLA possesses higher mechanical strength and is easier to process in comparison with other biopolymers and, additionally, is naturally degraded in soil or compost [7,8] in products completely assimilated by microorganisms [2]. Despite such strengths, PLA toughness, thermal stability, and mechanical properties are still limited when compared with their petroleum-based counterparts [9]. Despite its strong resistance, it is very brittle (elongation at break lower than 10%) [10] and another important shortcoming limiting its practical application is the relatively slow crystallization rate [4]. In this regard, to overcome these drawbacks, the addition of plasticizers, blending [11], copolymerization [12], as well as the development of micro- or nano-composites have been often employed as promising strategies to enhance the mechanical performance of PLA while preserving its transparency, which are both useful properties in the packaging sector [13,14].

Nowadays, food packaging is in constant development in order to meet the consumer and industry demands that trend towards minimally processed food products with prolonged shelf-life and controlled quality [15]. The packaging is known as the cornerstone of the food processing industry as almost 100% of the food and drinks that we buy and consume are packaged at a higher or lower level [16,17]. Packaging functions are mainly related to preservation and protection of the food, maintaining its quality and safety as well as the reduction of food waste [16]. Therefore, the only way to respond to this demand is by developing improved packaging concepts to guarantee food safety, quality, and traceability. In this sense, the development of active food-packaging can represent a big step forward to provide additional functions that prevent food waste in comparison with traditional non-active (passive) materials.

Food-borne pathogens are of utmost concern in food safety [18]. Each year, millions of people get sick or even die due to the ingestion of unsafe food and water, mainly caused by bacteria, viruses, and parasites [19], and as a direct consequence, antimicrobial active-packaging materials are attracting the attention from food and packaging industries [20]. Nowadays, the introduction of silver particles (Ag NPs) into polymers able to imprint antimicrobial properties, is widely accepted as silver is considered a potent broad-spectrum antimicrobial agent non-toxic to human cells and with proven long-lasting biocidal activity, high temperature stability, and low volatility [21,22,23,24]. Therefore, Ag NPs have been approved for use in food-contact polymers in the USA and the European Union, after previous consideration of the use conditions [25]. Recently, synthesis under eco-friendly conditions of silver (Ag) and gold (Au) nanoparticles have been gaining interest for researchers [25,26], as most common reducing agents employed for the preparation of nanoparticles are NaBH_4_ (sodium borohydride), citrate, or ascorbate, which are associated with environmental toxicity or biological hazards. In this context, chitosan is one of the most used biopolymers, thanks to their large amount of free amino and hydroxyl groups, and under the proper thermal conditions can be employed as reducing and stabilizing agents for the synthesis of Ag and Au nanoparticles preventing their aggregation [27,28,29,30,31,32,33]. In proper thermal conditions, the hydroxyl groups are converted to carboxyl groups by air oxidation, which reduces the silver ions [34].

With this background, in this study eco-friendly silver nanoparticles synthesized with a green protocol, using non-toxic biodegradable chitosan as the reducing agent, have been employed for obtaining plasticized PLA nanocomposites suitable for packaging applications thanks to its biocide properties. The aim of the present work is to study in detail the morphological, mechanical, thermal, and antibacterial properties as well as the crystallinity and the degradation profile in composting conditions of degradable plasticized PLA nanocomposites containing chitosan-based silver nanoparticles, in order to assess the prospective approach offered by these new systems. Nanocomposites have been prepared by melt compounding in a twin-screw extruder and their mechanical and thermal responses have been related to the nanoparticle content. The oligomeric lactic acid employed as a plasticizer agent together with the chitosan-based silver nanoparticles imposes enhanced ductility, toughness, and antibacterial activity to the developed nanocomposites.

## 2. Materials and Methods

### 2.1. Materials

Polylactic acid (PLA3051D, 3% of D-lactic acid monomer, molecular weight 142 × 10^4^ g/mol, density 1.24 g/cm^3^) and Lactic acid oligomer (Glyplas OLA8, ester content >99%, density 1.11 g/cm^3^, viscosity 22.5 mPa.s, molecular weight 1100 g/mol) were supplied from NatureWorks LLC (Minnetonka, MN, USA) and Condensia Química SA (Barcelona, Spain), respectively. Silver nitrate (AgNO_3_) and Chitosan from shrimp shells with a deacetylation degree >75% were purchased from Sigma-Aldrich (St. Quentin Fallavier, France). Acetic acid and sodium hydroxide were purchased from Fluka (Seelze, Germany).

### 2.2. Synthesis of Based Chitosan Silver Nanoparticles

Chitosan-based silver nanoparticles (AgCH-NPs) were synthesized by a method described elsewhere [33,35]. Typically, 4.5 mL of a solution of 52.0 mM of AgNO_3_ and 10 mL of a solution of chitosan of concentration 6.92 mg/mL in 1% acetic acid were mixed and heated at 95 °C under stirring for 12 h. The color of the mixture changed from colorless to yellow and, finally, to brownish which indicates the formation of the nanoparticles. The dispersions obtained were dialyzed for three days against distilled water using dialysis membranes with a MWCO of 12,000–14,000 Da. AgCH-NPs were then isolated by lyophilization.

### 2.3. Preparation of Oligomeric Lactic Acid Plasticized Poly(Lactic Acid) Nanocomposites Containing AgCH-NPs (PLA/OLA AgCH-NPs Nanocomposites)

Appropriate amounts of PLA, AgCH-NPs, and OLA were mixed in a microextruder equipped with two twin conical co-rotating screws (Thermo Scientific MiniLab Haake Rheomex CTW5). PLA pellets and OLA were previously dried overnight at 80 °C in order to avoid the presence of moisture. AgCH-NPs were dried at 40 °C for 4 h. PLA was first processed in the MiniLab mixer at 180 °C and a rotation speed of 100 rpm; after 2 min OLA and AgCH-NPs were added once PLA had reached the melt state to avoid oligomer and nanoparticle thermal degradation. The total time of mixing was 3 min. Films were then obtained by processing blends by compression molding at 180 °C in a hot press (Collin P-200-P, Collin Lab & Pilot Solutions GmbH, Maitenbeth, Germany). Blends were kept between the hot plates at atmospheric pressure for one minute at 180 °C until reaching the melt state. Then, they were submitted to the following pressure cycles, 5 MPa for one minute at 180 °C and then cooling down to room temperature at 5 MPa for one minute. The different obtained formulations are gathered in Table 1.

### 2.4. Characterization Techniques

A Philips XL30 scanning electron microscope (SEM, Philips, Mahwah, NJ, USA), with an accelerating voltage of 10 kV, a work distance of 10–15 mm was used to record SEM micrographs of samples cross-section and observe changes produced by the different amount of filler (AgCH-NPs). Nanocomposites were cryo-fractured with the aid of liquid N_2_ and each specimen was gold-coated (~5 nm thickness) in a Polaron SC7640 Auto/Manual Sputter (Quorum Technologies LTD, Kent, UK).

Fourier transmission spectra (FTIR) were recorded for all the samples using a Spectrum-Two (Perkin Elmer) spectrometer between 650 and 4500 cm^−1^ spectral range with a 4 cm^−1^ resolution and an attenuated total reflectance (ATR) cell. A background spectrum was acquired before every sample and all samples were vacuum-dried prior to measurement.

Thermal transitions and stability of neat PLA/OLA, as well as obtained nanocomposites with different amounts of AgCH-NPs, were studied by differential scanning calorimetry (DSC) and thermogravimetric analysis (TGA) in a DSC Q2000 and TA-Q500 apparatus both from TA Instruments (New Castle, DE, USA), respectively. In the DSC, samples were heated from −60 °C up to 180 °C at 10 °C /min under an N_2_ atmosphere (flow rate of 50 mL/min). Glass transition temperatures (T*_g_*), calculated as the midpoint of the transition, melting temperatures (T*_m_*), cold crystallization (*ΔH_cc_*), and melting enthalpies (*ΔH_m_*) were calculated by analyzing the thermograms in the TA Universal Analysis software. The degree of crystallinity (X*_c_*%) was therefore obtained from Equation (1) using 93.6 J/g as crystallization enthalpy value for pure crystalline PLA (*ΔH^0^_m_*) [36] W*_f_* represents the weight fraction of pure PLA present in the sample (*W_f_* = 1 − 0.2 − *m_f_*, where *m_f_* is the weight fraction of the nanoparticles in the nanocomposite).

(1) X*_c_*% = 100 × ((*ΔH_m_* − *ΔH_cc_*)/*ΔH^0^_m_*) × (1/W*_f_*)

Tensile tests were carried out on an Instron instrument (Instron, Norwood, MA, USA). equipped with a 50 N load cell, operated at room temperature and at a crosshead speed of 10 mm/min (strain rate). The initial length between clamps was set at 10 mm, samples of 6 mm width and ~100 µm of average thickness were measured and results from five to ten specimens were averaged. Young’s modulus (slope of the curve from 0–2% deformation), maximum stress, ultimate tensile strength, and elongation at break were calculated.

To study the crystalline structure of the plasticized PLA nanocomposite films, a Bruker D8 Advance X-ray diffractometer (Bruker Scientific LLC, Billerica, MA, USA) equipped with a CuKα (λ = 0.154 nm) source was employed. Samples were mounted on an appropriate holder and scanned between 2° and 60° (2θ) with a scanning step of 0.02°, a collection time of 10 s per step, and 40 kV of operating voltage to obtain X-ray diffraction patterns (XRD). Percentages of crystallinity were calculated with the aid of PeakFit software (Systat Software Inc. V4.12, San Jose, CA, USA) by a fitting process using a deconvolution method.

Antimicrobial activity of the prepared nanocomposites was determined following the E2149-13a standard method of the American Society for Testing and Materials (ASTM) (West Conshohocken, PA, USA) [37] against *Staphylococcus aureus (S. aureus,* ATCC 29213) and *Escherichia coli (E. coli*, ATCC 25922) bacteria. Each nanocomposite was placed in a sterile falcon tube and then 10 mL of the bacterial suspension (ca. 10^6^ colony forming units (CFU)/mL) were added. Falcon tubes with only the inoculum and neat plasticizer PLA were also prepared as control experiments. The samples were shaken at room temperature at 150 rpm for 24 h. Bacterial concentrations at time 0 and after 24 h were calculated by the plate count method. The contact killing experiment was done per triplicate on different days and the plate counting by duplicate.

The biodegradation test for all the samples was conducted under aerobic composting conditions in a laboratory-scale reactor following the ISO-20200 standard. [38] Briefly, samples were cut into square geometries of 15 × 15 mm and buried 4–6 cm in depth inside reactors containing solid biodegradation media −10 wt% of compost (Compo GmbH, Münster, Germany), 30 wt% of rabbit food, 10 wt% of starch, 5 wt% of sugar, 1 m wt% of urea, 4 wt% of corn oil, 40 wt% of sawdust and water in a 45:55 wt% ratio, and incubated at 58 °C for 40 days. Samples were kept into textile meshes to allow easy removal from the composting medium, while when buried access of microorganisms and moisture was ensured. Water was periodically added to the reaction containers to maintain the relative humidity in the medium, and the aerobic conditions were guaranteed by the regular mixing of the compost medium. Samples were recovered from the disintegration medium at different time intervals (7, 17, 21, 28, 36, and 44 days), washed with distilled water, and dried in an oven at 37 °C until constant weight. The mass loss weight % was calculated by normalizing the sample weight at different incubation times to the initial weight value. Photographs were taken from samples once extracted from the composting medium. The test was done at least with two samples of each composition.

## 3. Results

### 3.1. Microstructure and Morphology of the PLA/OLA AgCH-NPs Nanocomposites:

**Figure 1:**
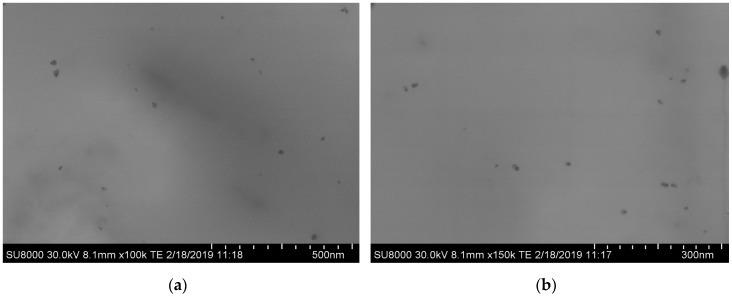
Scanning electron microscopy (SEM) images of AgCH-NPs at different magnifications, (**a**) ×100,000 and (**b**) ×150,000.

The morphology of AgCH-nanoparticles and PLA/OLA AgCH-NPs nanocomposites was studied by scanning electron microscopy (SEM). Figure 1 shows representative SEM micrographs of the synthesized AgCH-NPs, revealing a spherical morphology with diameters of ~8 ± 1 nm in agreement with previously reported values for similar procedures [35].

**Figure 2:**
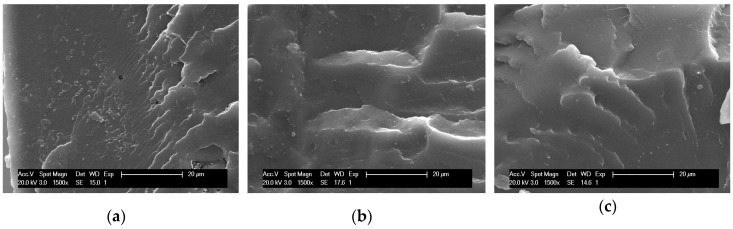

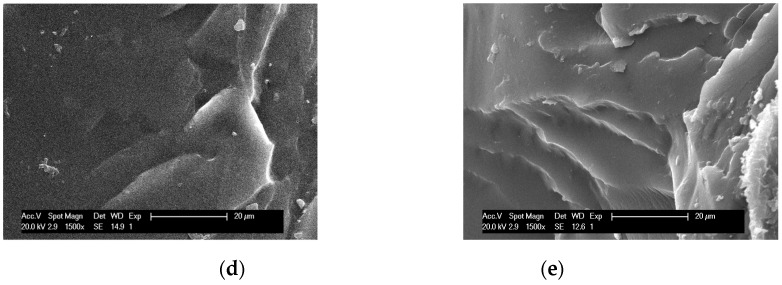
SEM images of the cross-section of PLA/OLA and PLA/OLA AgCH-NPs reinforced nanocomposites at ×1500 magnification, with different nanoparticles concentration. (**a**) PLA/OLA, (**b**) 0.5 wt% AgCH-NPs, (**c**) 1 wt% AgCH-NPs, (**d**) 3 wt% AgCH-NPs, (**e**) 5 wt% AgCH-NPs.

Figure 2 depicts scanning electron microscopy (SEM) images of the cross-sectional areas of neat PLA/OLA matrix and nanocomposites with 0.5 wt%, 1 wt%, 3 wt%, and 5 wt% of AgCH-NPs. Clearly, the incorporation of AgCH-NPs into the polymer matrix induces a change in the morphology of the cross-section surface. The unfilled material shows more uniform and plane fracture while for nanocomposites a surface with shear-yield and plastic deformation in different directions can be observed. The rougher surface morphology into the nanocomposites is homogeneously distributed along the cross-section and no large agglomerations of nanoparticles seem to be present. Additionally, voids and more stratified structures appeared and increased with the AgCH-NPs content probably due to more restricted mobility (higher rigidity) of the polymer chains at the interface with the nanoparticles.

**Figure 3:**
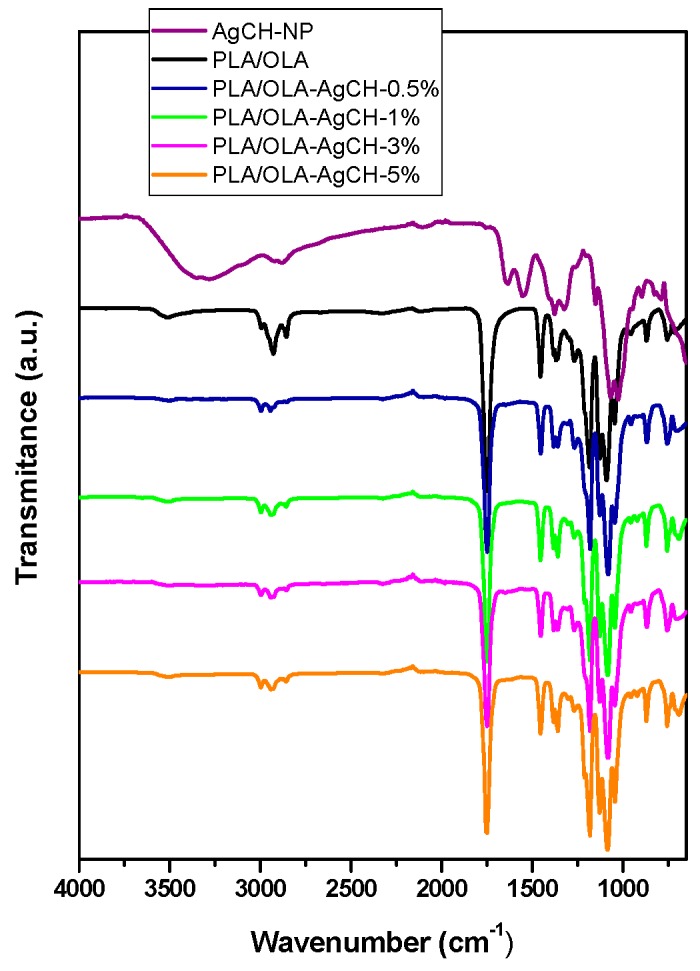
Fourier transmission spectra (FTIR) of PLA/OLA, AgCH-NPs and PLA/OLA AgCH-NPs reinforced nanocomposites.

FTIR spectra of PLA/OLA, AgCH-NPs and PLA/OLA AgCH-NPs reinforced nanocomposites are shown in Figure 3, where characteristic bands of PLA/OLA and AgCH-NP can be identified. Neat PLA/OLA and nanocomposites show a weak band at 3518 cm^−1^ that can be attributed to the vibration of the hydroxyl groups in the terminal chain of PLA/OLA. At 1748 cm^−1^ there is a carbonyl group stretching vibration band, and at 1454 cm^−1^, 1383 cm^−1^, and 1366 cm^−1^ appears –CH_3_ groups, -CH deformation, and asymmetric bands. The –C-O- stretching bands appear at 1180 cm^−1^, 1130 cm^−1^, and 1085 cm^−1^. AgCH-NPs spectrum exhibits the characteristics absorption bands of chitosan at 1639 cm^−1^ (Amide I), 1551 cm^−1^ (Amide II), and 1324 cm^−1^ (Amide III). These bands are not detectable in the nanocomposite’s spectra due to the low percentage of nanoparticles in the nanocomposites and the weakness of these bands.

**Figure 4:**
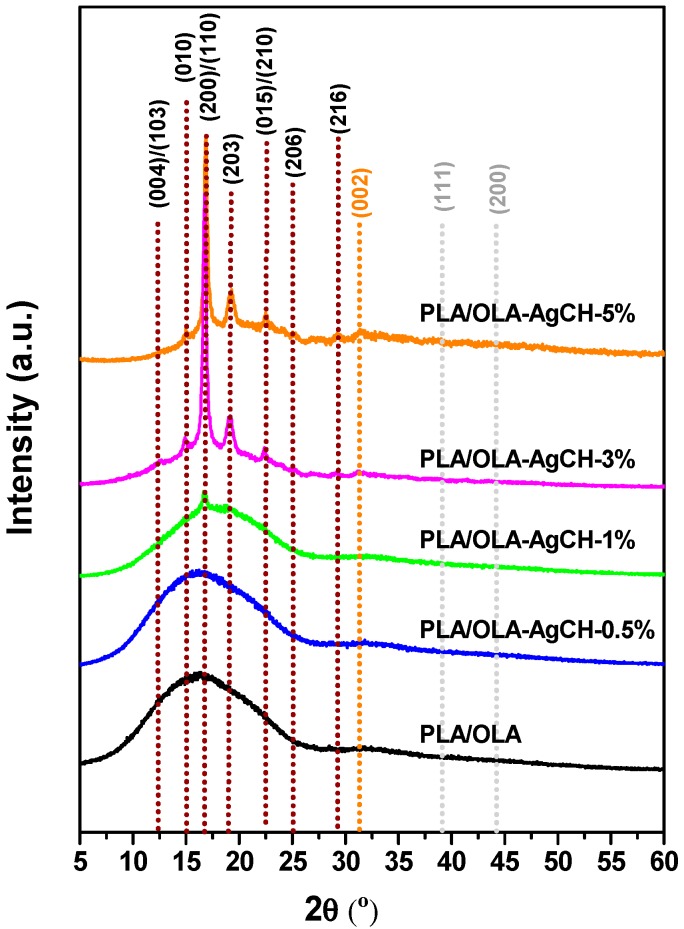
XRD patterns of PLA/OLA and PLA/OLA AgCH-NPs reinforced nanocomposites.

XRD patterns were recorded for all the samples and are shown in Figure 4 (see supporting information Figure S1 for AgCH-NPs XRD spectrum). Neat PLA/OLA exhibits a broad reflection indicative of its amorphous nature. The addition of 0.5 wt% of AgCH-NPs did not produce a considerable difference in the PLA/OLA matrix crystalline structure; however, the addition of 1 wt% of AgCH-NPs starts to induce crystallinity to the PLA/OLA matrix as evidenced by the appearance of a diffraction peak at 2θ = 16.7° corresponding to (110/200) planes of PLA. With increasing the AgCH-NPs content, this peak characteristic of the α and α′ type crystals become more evident. Additionally, in samples with 3 wt% and 5 wt% of AgCH-NPs loads, new peaks at 2θ = 15° and 2θ = 19.2° corresponding to (010) and (203) plane reflections of PLA chains belonging to α or α′ type crystals were visible, what is indicative of the nanoparticles nucleating effect [39,40].

Besides these diffraction peaks, samples with contents of 3 and 5 wt% of AgCH-NPs, start to exhibit reflections at around 32° (002), 38° (111), and 44° (200) corresponding to the presence of AgCH-NPs (see Supporting Information Figure S2) being a clear evidence of their presence into the nanocomposites. Interestingly, with increasing the AgCH-NPs content to 5 wt%, reflections from (010), (200/110), and (203) planes very slightly shifted to higher 2θ, and intensity of peaks at 15° and 22.4° corresponding to (010) and (015) crystalline planes of PLA, increased (Table 2). This fact can be indicative of a higher degree of order in the structure with increasing the AgCH-NPs content to 5 wt% from more distorted orthorhombic crystals (α′ form) to more perfect crystals (α form). Calculated interplanar distance from Braggs’ law evidenced lower distance between planes in the sample with the highest AgCH-NPs content (Table 2) [41]. It is also possible to note that small diffraction peaks at 12.6° and 25.3° appeared in 1, 3, and 5 wt% of AgCH-NPs containing samples that are assigned to the reflections of (004/103) and (016) planes of crystalline PLA respectively [42,43].

### 3.2. Thermal Analysis

**Figure 5:**
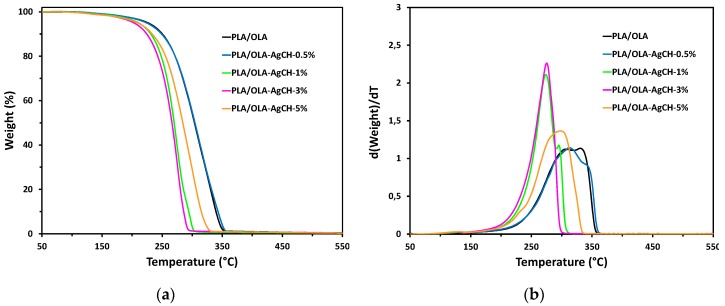
Thermogravimetric curves of neat PLA/OLA and its nanocomposites with different AgCH-NPs content: (**a**) Weight loss vs. temperature curves; (**b**) derivative curves.

In order to study the effect of the addition of AgCH-NPs over the PLA/OLA matrix thermal stability as well as over the thermal degradation mechanism, thermogravimetric analysis was conducted under nitrogen atmosphere. Figure 5 shows weight loss vs. temperature thermogravimetric curves (Figure 5a) and their corresponding derivative curves (Figure 5b), for neat PLA/OLA and AgCH-NPs loaded PLA/OLA nanocomposites. Table 3 collects temperatures at different percentages of weight loss for each sample.

It is worth noticing that derivative thermogravimetric curves (DTG) of nanocomposites containing 1 and 3 wt% of AgCH-NPs are sharper than the rest (neat matrix and the other nanocomposites), which the main degradation peak presents a shoulder, evidencing a two-step degradation process. In general, the thermal stability of the samples seems to be dependent on the amount of AgCH-NPs. Maximum degradation temperature decreases between 20–56 °C with increasing AgCH-NPs content from 1 to 3 wt%. Interestingly, with the addition of 5 wt% of AgCH-NPs, the shape of the degradation curve changes significantly, with a temperature at maximum weight loss rate closer to the nanocomposites with 0.5 wt% of AgCH-NPs instead of the one with 3 wt% of AgCH-NPs. This phenomenon could be related with the high amount of AgCH-NPs that can affect the mass transport barrier, resulting in more intricate paths for the volatile products (i.e., low molecular weight OLA plasticizer) to scape during thermal decomposition and therefore, promoting slightly better thermal resistance for sample with AgCH-NPs loading of 5 wt% [44,45]. Thus, in general, TGA results show that the addition of low amounts of AgCH-NPs (0.5 wt%) did not significantly affect the thermal degradation of the PLA/OLA matrix, while amounts closer to 5 wt% of AgCH-NPs could even better prevent the thermal degradation of the matrix than the addition of 1–3 wt% of nanoparticles. Additionally, it is important to remark that no degradation occurred from room temperature to 180 °C, the range where nanocomposites were processed.

**Figure 6:**
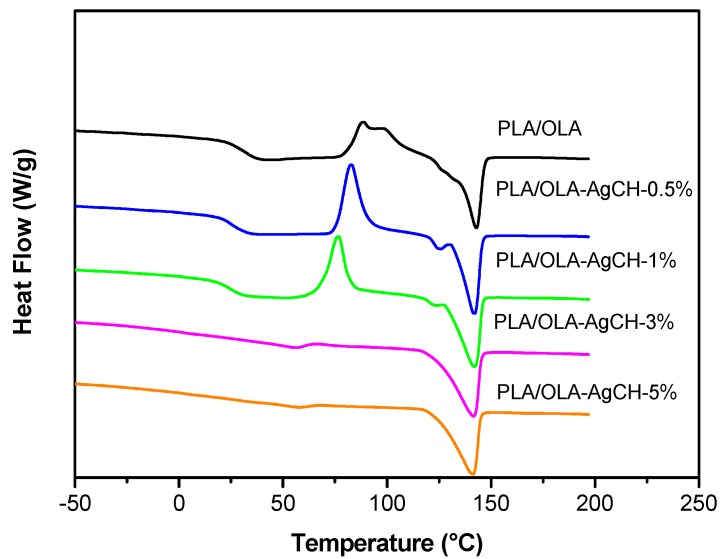
Differential scanning calorimetry (DSC) curves of neat PLA/OLA and its nanocomposites with different AgCH-NPs content.

Differential scanning calorimetry (DSC) was employed to study the glass transition, melting, and crystallization of plasticized PLA and its silver-containing nanocomposites. Figure 6 shows the thermograms of neat PLA/OLA and its AgCH-NPs nanocomposites. Glass transition (T*_g_*), cold crystallization (T_cc_, *ΔH_cc_*), and melting temperatures and enthalpies (T*_m_*, *ΔH_m_*), as well as crystallinity values (X*_c_*%) are summarized in Table 4. All the samples showed a single glass transition temperature that is indicative of good miscibility between polymer matrix and plasticizer as no evidence of T_g_ from free OLA was observed. The addition of AgCH-NPs into PLA/OLA resulted in significant differences over T*_g_* values with respect to the neat matrix. The addition of 0.5–1 wt% of AgCH-NPs produced a significant decrease in T*_g_* values from 32 °C to 24–25 °C. This reduction due to the addition of nanoparticles can be related to the higher mobility of the polymer macromolecules due to an increase in the free volume of the matrix probably due to the plasticizer. On the contrary, the addition of 3–5 wt% of AgCH-NPs increased the T*_g_* towards typical values of non-plasticized PLA probably due to the presence of high content of nanoparticles within the PLA plasticized matrix, that hinders the movements of the macromolecular chains in agreement with TGA results [46].

**Figure 7:**
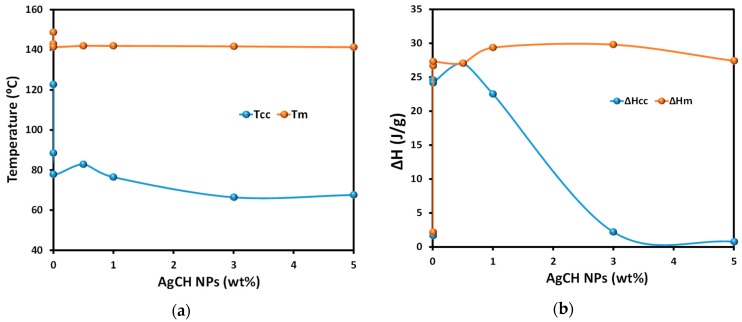
(**a**) T*_m_* and T*_cc_*, and (**b**) ΔH_cc_ and ΔH_m_ evolution at different AgCH-NPs contents in the PLA/OLA nanocomposites.

Accordingly, and as can be seen in Figure 7, T*_cc_* values decreased as well as the area under the peak (*ΔH_cc_*) with the addition of AgCH-NPs that acts as nucleus promoting/favoring crystallization of PLA/OLA at lower temperatures. Moreover, the cold crystallization in samples with 3 and 5 wt% of AgCH-NPs is barely visible compared to the samples with lower AgCH-NPs content, which agrees with their higher crystallinity just after processing as confirmed by XRD. T*_m_* values did not show notable differences when nanoparticles are present, and all the samples showed a broad transition with multiple shoulders which appear well-defined as two individual peaks in samples with 0.5 and 1 wt% of AgCH-NPs contents. As previously reported for some research, this phenomenon can be attributed to the melting of different crystals (α and α′) mainly formed during heating in DSC analysis as the total enthalpy (*ΔH_Total_*) of the continuous transitions for these samples in the range between crystallization and melting is near zero. The fact that *ΔH_Total_* value in 1 wt% of AgCH-NPs sample is slightly higher is in agreement with its higher crystallinity compared to neat PLA/OLA and nanocomposite containing 0.5 wt% of AgCH-NPs. In correlation with XRD results, the clearly visible first melting peak can be due to the presence of α′ form just after processing, or crystallites with different lamellar thickness. In contrast, it is clear that higher AgCH-NPs content (3–5 wt%) induces crystallinity during processing as evidenced by the low *ΔH_cc_* values and subsequent melting peak.

### 3.3. Mechanical Properties

**Figure 8:**
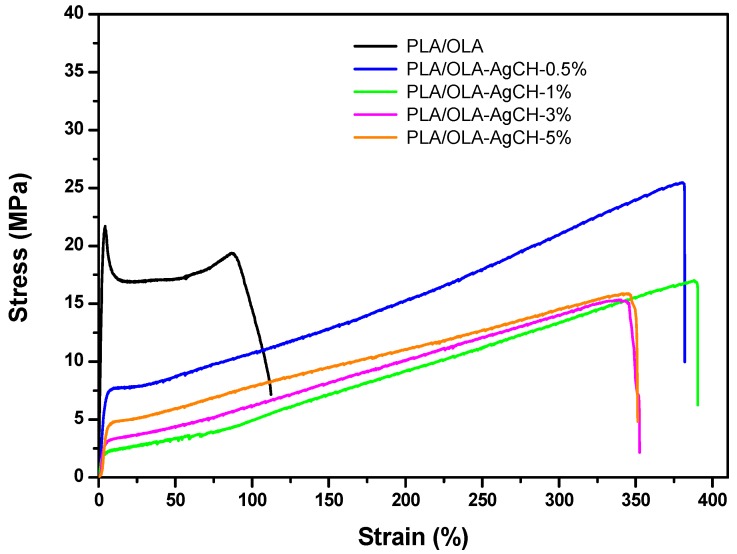
Representative tensile stress vs. strain curves obtained for neat PLA/OLA and PLA/OLA AgCH-NPs nanocomposites.

The characteristic mechanical behavior of all processed samples is shown in Figure 8 and values are summarized in Table 5. Neat PLA/OLA showed low elongation at break, (ε), (neat PLA/OLA ε ~108%; AgCH-NPs nanocomposites ε ~338–371%) and high Youngs’ or elastic modulus, (E), (neat PLA/OLA E = 783 MPa; AgCH-NPs nanocomposites E ~88–256 MPa) compared to nanocomposites. All the nanocomposites possessed higher elongation at break while a reduction in the elastic modulus and maximum tensile strength (σ_max_) was observed similarly as previously reported in other studies [41]. Interestingly, the best balance of mechanical behavior into the nanocomposites was obtained for the least amount of AgCH-NPs tested (0.5 wt%) having the highest Youngs’ modulus and maximum tensile strength of the whole nanocomposites while retaining similar elongation at break. This sample (0.5 wt% of AgCH-NPs) is totally amorphous, therefore, the low amount of filler helps to retain mechanical properties closer to the neat PLA/OLA matrix that also possesses the lowest crystallinity. Thus, it seems that the nanoparticles did not disturb the chain mobility allowing for an increased elongation at break also enhanced with the loss of crystallinity of this system. In the rest of the nanocomposites, AgCH-NPs started to induce crystallinity producing an increase of the Youngs’ modulus with the % of crystallinity and, therefore, with the number of nanoparticles. This fact can also be related to the observation from XRD results that evidenced the presence of crystals with a higher perfection degree into the 5 wt% of the AgCH-NPs sample, in comparison to the crystalline samples with a lower number of nanoparticles. In this regard, it is well known that the toughness of semicrystalline polymers is strongly influenced by the variation of crystalline structure and therefore by the solid density, affecting not only the elastic modulus but also the elongation at break. In the nanocomposites, toughness decreased with crystallinity, being lower for nanocomposites with 1–5 wt% of AgCH-NPs that possess some crystallinity in comparison with 0.5 wt% AgCH-NPs nanocomposites.

### 3.4. Antibacterial Activity

**Figure 9:**
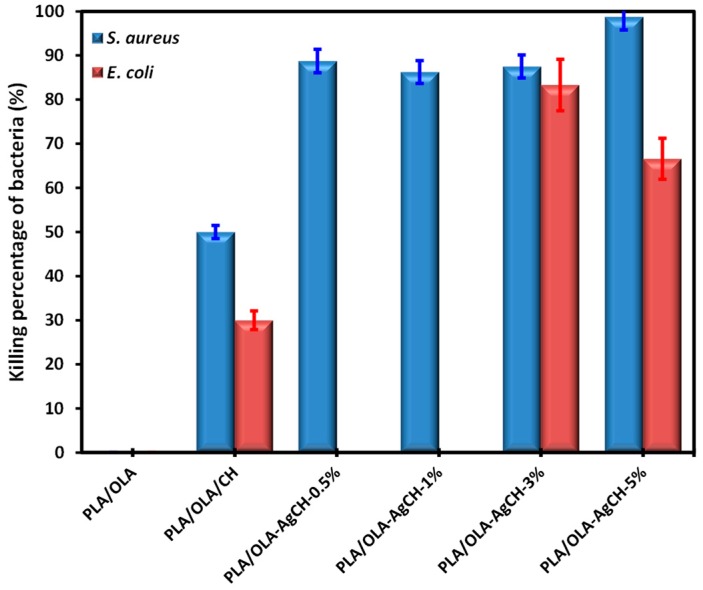
Percentage of killing bacteria for the different nanocomposites.

The effectiveness of these nanocomposites against Gram-positive *S. aureus* and Gram-negative *E. coli* bacteria was evaluated by the ASTM standard method of [37]. The chitosan is well-known to have antimicrobial activity depending on its structure and molecular weight. For comparison purposes, one nanocomposite using chitosan at in-between compositions (2%) was obtained by the same procedure. Figure 9 displays the antibacterial activity of all nanocomposites represented as the percentage of bacteria kill, which was calculated by the difference between the CFU after contact with control substrates (PLA/OLA and none) and CFUs after contact with the polymeric nanocomposites.

When chitosan is introduced it confers activity to the matrix, but this effect is more evident when AgCH-NPs are introduced. The behavior against Gram-positive is more powerful than against Gram-negative since the latter presents the outer lipid cell wall, which is more difficult to destroy. In the case of AgCH-NPs low content, the nanocomposites are not effective against *E. coli* but are against *S. aureus* bacteria.

### 3.5. Disintegration under Composting Conditions of PLA/OLA AgCH-NPs Nanocomposites:

**Figure 10:**
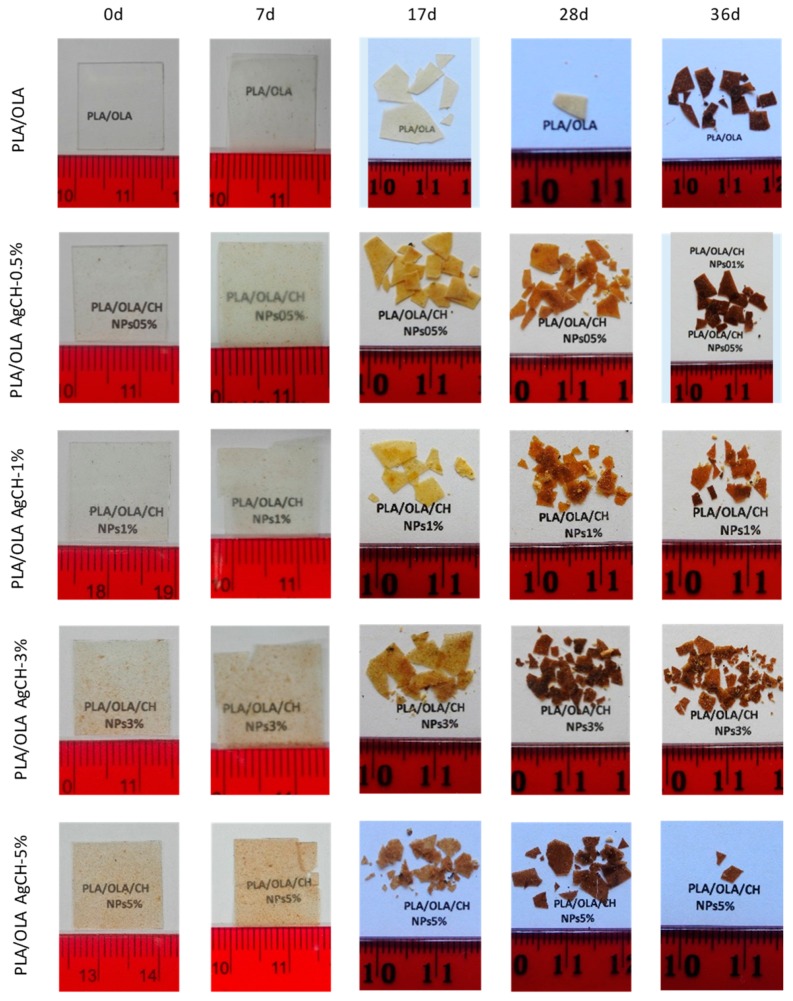
Disintegrated samples under composting conditions.

In order to evaluate the ability of PLA/OLA and PLA/OLA AgCH-NPs active nanocomposites to undergo disintegration, firstly, a visual examination of samples at different times when subjected to composting conditions was performed and results are collected in Figure 10. After seven days of incubation, nanocomposites containing 1–5 wt% of AgCH-NPs already exhibited fractures, while at 17 days of incubation all samples broke down and changed color, becoming totally opaque. In general, a change in the color is due to a variation in the refraction index due to water absorption and/or presence of products formed by the hydrolytic process being a clear indicator of degradation processes occurring [47,48].

**Figure 11:**
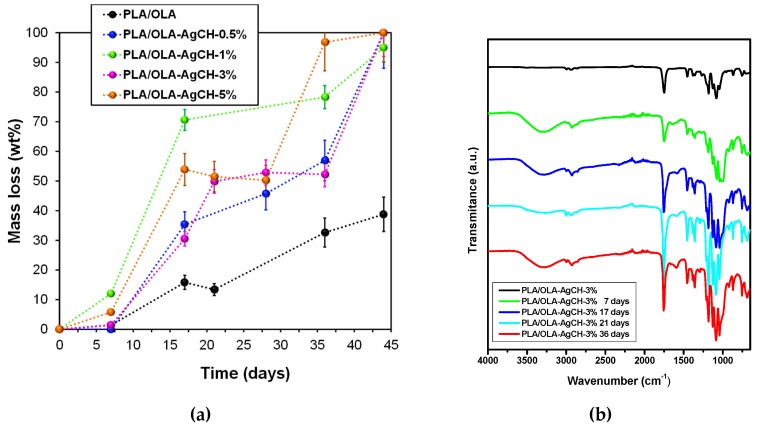
(**a**) Weight loss percentage values of PLA and PLA/OLA AgCH-NPs nanocomposites at different incubation times under composting conditions. (**b**) Infrared spectra of PLA/OLA nanocomposites containing 3 wt% of AgCH-NPs, before composting and after different disintegration times.

All the samples were visibly disintegrated after 28 days. Thus, the nanocomposites containing 1–5 wt% of AgCH-NPs started to disintegrate faster than neat PLA/OLA and PLA/OLA containing a low number of nanoparticles (0.5 wt%). These results were confirmed by the weight loss values (Figure 11a) that remained almost constant for all the samples until seven days of incubation while, at 36 days, values near 50% and 80% were reached for nanocomposites containing 0.5 wt%, 3 wt%, and 1 wt%, 5 wt% of AgCH-NPs, respectively. Interestingly, all the nanocomposites degraded faster than the neat PLA/OLA matrix; this fact might be attributed to the release of Ag ions that can catalyze the disintegration of the samples accelerating the degradation process. Figure 11b shows, as an example, the FTIR spectra in the 650–4000 cm^−1^ range of sample PLA/OLA AgCH-3% at different composting times. The carbonyl group (–C=O) and –C–O group stretching from PLA and OLA are clearly visible at 1748 cm^−1^ and 1180 cm^−1^, respectively. After seven days of composting, a band, whose intensity increases over composting time, appeared at 1590 cm^−1^ due to carboxylate ions indicating the consumption of lactic acid by the microorganisms [49].

## 4. Conclusions

In this work, novel plasticized PLA nanocomposites containing 0.5 wt%, 1 wt%, 3 wt%, and 5 wt% of synthesized chitosan mediated silver nanoparticles (AgCH-NPs) were prepared by melt compounding in a twin-screw extruder and profusely characterized. The presented research depicts a new utilization of antimicrobial AgCH-NPs synthesized using nontoxic biodegradable chitosan as a reducing agent of silver nitrate. The AgCH-NPs obtained from a facile green synthesis method, clearly affected the crystal structure as well as the ability of neat PLA/OLA matrix chains to organize enhancing its crystallinity and lowering the cold crystallization temperature. In fact, as proven by XRD, nanoparticles induced a change in the lattice spacing (lowering the distance between crystalline planes) of the neat matrix, indicating the formation of crystals with higher perfection for the nanocomposite containing 5 wt% of nanoparticles. In accordance with XRD, a detailed analysis of DSC thermograms showed that the crystallinity degree of PLA/OLA was significantly affected when increasing the AgCH-NPs reaching values of ~37% for 3 wt% and 5 wt% of nanoparticle loads. A great enhancement in toughness and elongation at break was noticed for all the nanocomposites as compared with the neat PLA/OLA, while Youngs’ modulus did not change significantly upon the increase of AgCH-NPs wt%. In general, the properties of PLA were improved with the AgCH-NPs, mechanical and thermal properties showed balanced results despite the concentration of nanoparticles, and interestingly, the incorporation of even the smallest amount of AgCH-NPs increased significantly the disintegration rate under composting conditions. PLA/OLA AgCH-NPs nanocomposites exhibited antimicrobial activities against Gram-positive *S. aureus* and Gram-negative bacteria as compared to the pristine matrix. A dual mechanism of action combining the bactericidal effect of silver with the cationic effect of chitosan is probably the underlying and responsible mechanism for the enhanced antibacterial properties of the nanocomposites. The bactericide effect together with the enhanced degradation rate, ductility, toughness, and crystallinity of the developed nanocomposites as well as the possibility to be processed under the same conditions than neat PLA without thermal degradation, are good indications for their potential utilization in antimicrobial active food packaging applications.

## Figures and Tables

**Table 1:** Poly(lactic) acid/oligomeric lactic acid (PLA/OLA) AgCH-NPs formulations.

Sample	PLA (wt%)	OLA (wt%)	AgCH-NPs (wt%)
PLA/OLA	80	20	0
PLA/OLA-AgCH-0.5%	79.6	20	0.4
PLA/OLA-AgCH-1%	79.2	20	0.8
PLA/OLA-AgCH-3%	77.6	20	2.4
PLA/OLA-AgCH-5%	76	20	4

**Table 2:** Distance between planes of nanocomposites (1–5 wt% of AgCH-NPs) calculated from the most intensive diffraction peaks.

	2θ (Angle)			Distance between Planes		
Sample	(010)	(200)/(110)	(203)	d(A)	d(A)	d(A)
PLA/OLA AgCH1%	--	16.7	--	--	5.30	--
PLA/OLA AgCH3%	14.8	16.7	19.1	5.98	5.30	4.64
PLA/OLA AgCH5%	14.9	16.8	19.2	5.94	5.27	4.62

**Table 3:** Temperatures at different weight losses of neat PLA/OLA and the different formulations containing AgCH-NPs.

Sample	Temperature at Maximum Weight Loss Rates (°C)		Temperature at Different Weight Losses (°C)			
	T_max1_	T_max2_	T_5_	T_30_	T_50_	T_70_
PLA/OLA	306	331	227	284	304	321
PLA/OLA AgCH0.5%	312	341	224	285	304	322
PLA/OLA AgCH1%	273	296	211	258	270	279
PLA/OLA AgCH3%	275	--	203	253	266	276
PLA/OLA AgCH5%	303	--	210	268	284	299

**Table 4:** Thermal properties and crystallinity calculated from DSC scan for neat PLA/OLA and formulations containing AgCH-NPs.

Sample	T_g_	T_cc_	ΔH_cc_	T_m_	ΔH_m_	ΔH_Total_	X_c-DSC_ (%)	X_c-XRD_ (%)
PLA/OLA	32	88	25	143	27	2	2.8	--
PLA/OLA AgCH0.5%	25	83	27	142	27	0	0.0	--
PLA/OLA AgCH1%	24	76	23	142	29	6	9.2	3.3
PLA/OLA AgCH3%	50	66	2	142	30	28	38.0	26.2
PLA/OLA AgCH5%	53	68	1	141	27	26	37.5	21.9

**Table 5:** Mechanical properties for neat PLA/OLA and formulations containing AgCH-NPs.

Sample	E (MPa)	ε (%)	σ_max_ (MPa)	Toughness (MJ/m^3^)
PLA/OLA	783 ± 102	108 ± 6	23 ± 2	1.8 ± 0.1
PLA/OLA AgCH0.5%	256 ± 29	372 ± 26	23 ± 2	5.2 ± 0.7
PLA/OLA AgCH1%	88 ± 13	368 ± 32	16 ± 1	3.1 ± 0.6
PLA/OLA AgCH3%	123 ± 36	369 ± 50	16 ± 2	3.3 ± 0.5
PLA/OLA AgCH5%	132 ± 29	338 ± 51	14 ± 3	3.1 ± 0.9

## Supplementary Materials

The following are available online at https://www.mdpi.com/2079-4991/10/1/22/s1, Figure S1: UV–vis absorption spectra of AgCH-NPs, Figure S2: XRD spectrum of AgCH-NPs.
