# Supplementary material for: Multifunctional PLA Blends Containing Chitosan Mediated Silver Nanoparticles: Thermal, Mechanical, Antibacterial, and Degradation Properties
Source: Nanomaterials (Basel). 2019 Dec 20;10(1):22. doi: 10.3390/nano10010022 (PMC7022492; doi:10.3390/nano10010022)
Supplement: Supplementary file 1 [file nanomaterials-10-00022-s001.pdf]

Multifunctional PLA/OLA blends containing Chitosan mediated Silver nanoparticles: thermal, mechanical, antibacterial and degradation properties.

Agueda Sonseca [1,2,4*], Salim Madani [3], Gema Rodríguez [1,2], Víctor Hevilla[1,2], Coro Echeverría [1,2], Marta Fernández-García [1,2], Alexandra Muñoz-Bonilla [1,2], Noureddine Chavef [3], Daniel López [1,2*]

1 MacroEng Group, Instituto de Ciencia y Tecnología de Polímeros, ICTP-CSIC, C/ Juan de la Cierva 3, 28006 Madrid, Spain
2 Interdisciplinary Plataform for "Sustainable Plastics towards a Circular Economy" (SUSPLAST-CSIC), Madrid, Spain
3 Laboratory of Applied Biochemistry, Department of Biology, Faculty of Sciences, University Ferhat Abbas, Setif 19000, Algeria
4 Current address: Instituto de Tecnología de Materiales, Universitat Politècnica de València (UPV), Camino de Vera s/n, 46022 Valencia, Spain

* Correspondence: agsonol@posgrado.upv.es (A.S), daniel.l.g@csic.es (D.L.);Tel.: +34 915622900

Supporting Information

Characterization of nanoparticles

Figure S1 shows the typical UV-vis absorption spectra of the resulting AgCH-NPs solution after lyophilisation of the reduced solution of silver nitrate with chitosan. The characteristic surface plasmon resolnance (SPR) band centred at about 420 nm is indicative of the formation of silver nanoparticles [1].

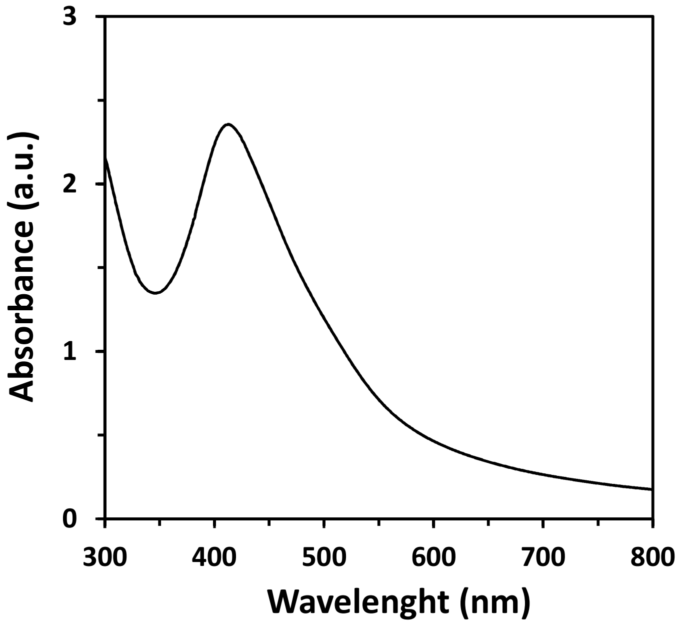

Figure S1. UV–vis absorption spectra of chitosan-based silver nanoparticles (AgCH-NPs) prepared by reducing $AgNO_3$ in a chitosan solution.

Figure S2 displays the XRD spectrum of silver NPs obtained by green chemistry method. It can be observed the characteristics peaks at 38º and 44º, which indicates face-centererd cubic Ag crystals (-fcc- JCPDS 04-783). Besides, three intense peaks at around 28º, 32º and 46º, which corresponds to (110), (111) and (211) of Ag_2O, are also identified (JCPDS 76-1393) [2].

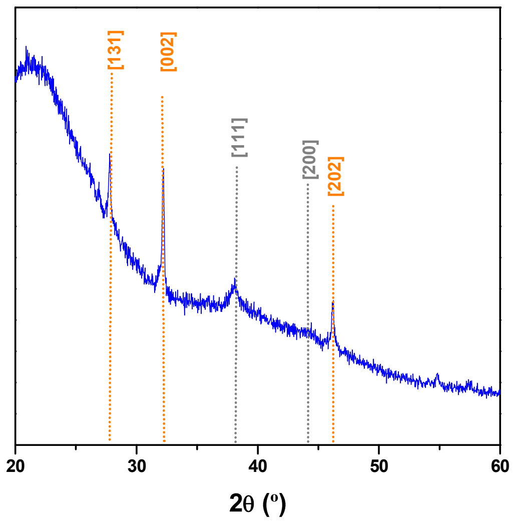

Figure S2. XRD spectrum of synthesized chitosan mediated AgCH-NPs.

References

1. Jia, X.; Ma, X.; Wei, D.; Dong, J.; Qian, W. Direct formation of silver nanoparticles in cuttlebone-derived organic matrix for catalytic applications. *Colloids Surfaces A Physicochem. Eng. Asp.* **2008**, *330*, 234–240.
2. Pawar, O.; Deshpande, N.; Dagade, S.; Waghmode, S.; Nigam Joshi, P. Green synthesis of silver nanoparticles from purple acid phosphatase apoenzyme isolated from a new source Limonia acidissima. *J. Exp. Nanosci.* **2016**, *11*, 28–37.
